# Asymmetric Parietal Cortical Atrophy in a Patient with *RAB39B* ‐Associated Parkinsonism

**DOI:** 10.1002/mdc3.70310

**Published:** 2025-08-26

**Authors:** Luca Gallo, Emna Ben Mansour, Silvia Nicolosi, Anna Pichiecchio, Simone Gana, Samia Ben Sassi, Silvano Cristina, Micol Avenali, Enza Maria Valente

**Affiliations:** ^1^ Department of Brain and Behavioural Sciences University of Pavia Pavia Italy; ^2^ Neurology Department National Institute of Neurology Tunis Tunisia; ^3^ Advanced Imaging and Artificial Intelligence Unit IRCCS Mondino Foundation Pavia Italy; ^4^ Neurogenetics Unit IRCCS Mondino Foundation Pavia Italy; ^5^ Neurorehabilitation Unit IRCCS Mondino Foundation Pavia Italy; ^6^ Parkinson Disease and Movement Disorders Unit IRCCS Mondino Foundation Pavia Italy; ^7^ Department of Molecular Medicine University of Pavia Pavia Italy

**Keywords:** parkinsonism, RAB39B, X‐linked disease

The *RAB39B* (Ras Analogue in Brain 39b) gene on the X chromosome encodes for a small GTPase protein involved in intracellular trafficking and mainly expressed in the central nervous system.^1^

Loss‐of‐function variants of *RAB39B* cause early‐onset parkinsonism with intellectual disability, predominantly in males.^2^ To date, 78 patients have been described, harboring 26 distinct variants^3^ (supplementary references). The imaging phenotype associated to this genetic condition has not been characterized in detail. We report a novel *RAB39B* pathogenic variant in a patient with late‐onset parkinsonism, paramagnetic deposits in the basal ganglia and left‐predominant parietal cortical atrophy.

## Case Report

The patient is a 57‐year‐old Caucasian male, born from unrelated parents, with three maternal uncles presenting intellectual disability, of whom one also with parkinsonism. Comorbidities include diabetes mellitus type 2 and systemic hypertension.

He had mild social skill impairment since childhood but completed secondary school. Parkinsonian symptoms appeared at age 50 years, with subtle resting tremor in the right upper limb and camptocormia. DAT‐SCAN showed bilateral reduced uptake in the right putamen/caudate nucleus. After 2 years, levodopa therapy was started (LEDD 300 mg), with good motor control.

We first evaluated the patient at age 52 years. Physical and neurological examination revealed mild craniofacial dysmorphisms (long and narrow face, large ears and broad forehead), resting, postural and kinetic tremor only on the right side, slight bradykinesia and ipsilateral reduced arm swing (Video 1). Neurocognitive evaluation revealed mild intellectual disability (IQ 53). Brain MRI at age 52 years showed bilateral paramagnetic deposits in the substantia nigra and basal ganglia, along with diffuse cortical atrophy predominantly involving the parietal lobes, with marked left‐sided asymmetry.

**VIDEO 1:** First neurological assessment. In this video, we can see the patient at his first neurological evaluation, at age 52 years. Mild resting tremor in the right hand can be observed, which is more pronounced during walking. There is also mild bradykinesia in the right side.

NGS‐based genetic analysis of a virtual panel of 68 genes related to parkinsonism identified a novel hemizygous truncating variant in *RAB39B* (NM_171998.4): c.202C>T; p.(Gln68*).

**FIG 1:**
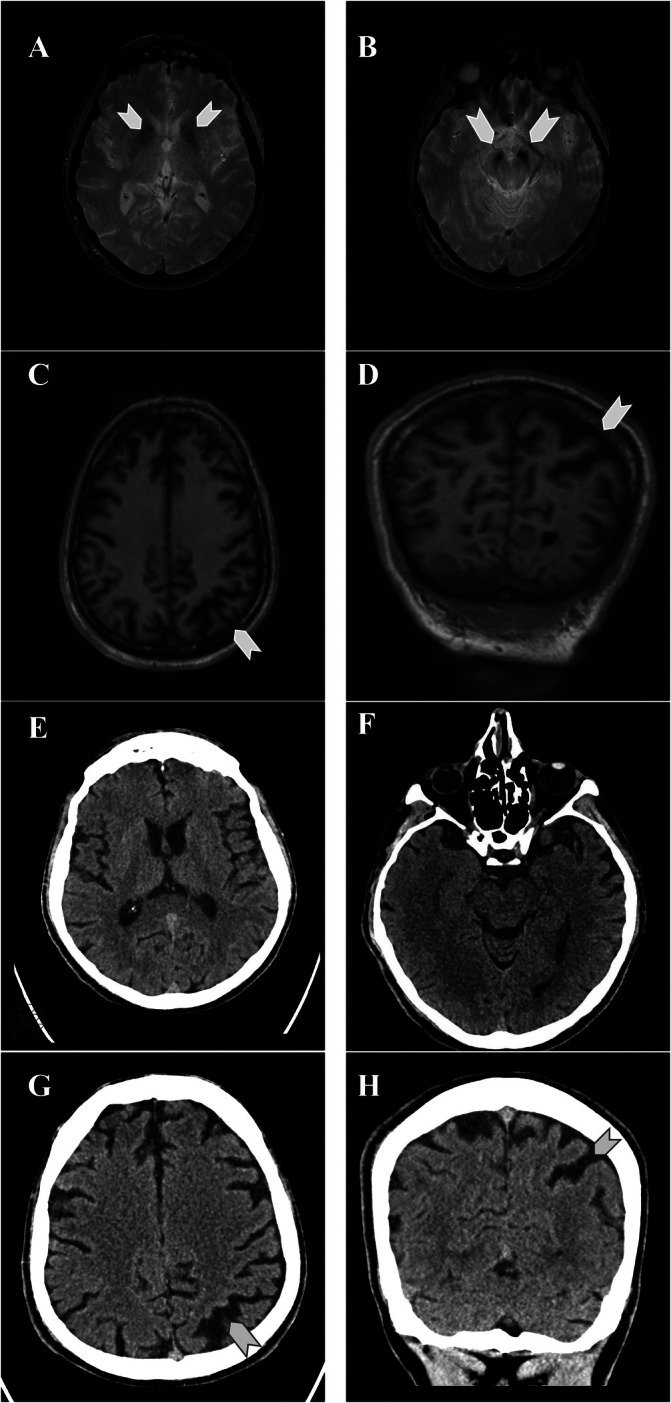
Brain MRI and CT of the patient at 57 years. Axial‐gradient‐recalled‐echo‐T2*‐weighted images demonstrate bilateral hypointensities in the substantia nigra (A) and globi pallidi (B), likely related to paramagnetic deposits. 3D‐T1‐weighted axial reconstruction (C) shows diffuse cortical atrophy mostly prevalent in the parietal regions. Parietal atrophy is also evident in the coronal section (D). SWI (susceptibility Weighted Imaging) sequence could not be obtained due to motion artifacts. Axial CT brain scan shows absence of calcifications in the basal ganglia (E) and substantia nigra (F). Cortical atrophy is visible on axial (G) and coronal reconstruction images (H).

Motor symptoms slowly worsened, with tremor becoming bilateral, and the patient progressively developed autonomic symptoms, mood disorders, dysphagia, pain, and sleep disturbances. At age 57 years, motor fluctuations occurred, with disabling tremor‐akinetic off periods, occasionally associated with freezing of gait (Video 2). LEDD was increased with limited motor benefit. The cognitive profile remained stable (Supplementary Table S1). A second brain MRI showed unmodified paramagnetic deposits and cortical atrophy. A concurrent CT scan excluded cerebral calcifications (Figure 1).

**VIDEO 2:** Neurological evaluation after 5 years, at age 57 years. The disease showed marked progression, with severely disabling tremor and worsening bradykinesia impairing agility tests. the patient still walks unassisted, with a positive pull test and preserved recovery. The patient is still able to walk without support. Pull test is positive with recovery.

## Discussion

Here we report a novel *RAB39B* truncating variant in a male presenting with mild intellectual disability, late‐onset parkinsonism and brain imaging abnormalities. The typical *RAB39B*‐associated phenotype features infantile‐onset non‐progressive intellectual disability associated with parkinsonism;^2^ epilepsy, autism spectrum traits and craniofacial dysmorphism can also occur.^4^, ^5^, ^6^ Parkinsonian signs usually manifest early (mostly in the third decade), yet the spectrum of ages at onset is broad and few cases have been reported with adult onset.^3^

The neuroimaging phenotype of *RAB39B*‐related parkinsonism has been described only in 26 patients, with heterogeneous outcome (Table 1). In 14 patients, brain MRI was reported as normal. However, this finding should be interpreted with caution, as several individuals underwent imaging in the first or second decade of life, when alterations may not have developed yet. Furthermore, all but one negative case did not undergo a CT scan, thus the presence of subtle calcifications cannot be ruled out with certainty. Six patients had basal ganglia calcifications which were demonstrated by CT scan, and confirmed in many cases by the detection of hypointense signal at MRI sequences sensitive to paramagnetic and diamagnetic compounds, such as SWI (Susceptibility Weighted Imaging) and T2 GRE (Gradient‐Recalled Echo). Similar hypointensities of the basal ganglia and, often, of the substantia nigra were reported in a further six patients, using a range of MRI sequences with variable sensitivity in detecting paramagnetic and diamagnetic compounds. Interestingly, in two of these patients, including the one reported here, CT scan excluded the presence of brain calcifications, suggesting that the observed hypointensities could result from deposition of paramagnetic compounds such as iron. The underlying pathology still remains uncertain in the other four cases.

**TABLE 1:** Summary of available neuroimaging findings in *RAB39B*‐mutated subjects

Reference	Patients	Brain MRI	Brain CT scan
Giannandrea et al^6^	II‐3 (M, 52y), III‐3 (M, 13y)	Both: normal	‐
Vanmarsenille et al^11^	II.1 (M,6.5) AV1, (M, 22y), KM1 (M, 6.5y)	All: normal	‐
Wilson et al^12^	II:1 (M, adult age)	Mild hypointensity of SN (T2)	‐
	II:2 (M, adult age)	Normal	‐
El‐Hattab et al^13^	Family 4 (M, 12y), Family 6 (F, 11y), Family 7 (F, 0.25y)	All: normal	‐
Lesage et al^14^	M, 39y	Normal (repeated twice)	‐
Güldner et al^15^	M, 42y	Hypointensity of SN and BG (T2*)	‐
Shi et al^16^	II‐2 (M, 58y), III‐1 (M, 20y)	Both: hypointensity of bilateral GP (SWI)	Calcifications of BG
Ciammola et al^17^	Family 1, II‐3 (M, 67y)	Hypointensity of SN and GP, milder hypointensity of red nuclei, putamen, and pulvinar (GRE, SWI)	Moderate calcifications of GP
	Family 1, II‐1 (M, adult age)	‐	Moderate calcifications of GP
	Family 2, IV‐2 (M, 49y)	Hypointensity of SN and GP, milder hypointensity of red nuclei, putamen, and pulvinar (GRE, SWI)	No calcifications
Ballout et al^18^	Case 3 (F, 2y)	Normal	‐
Santoro et al^19^	M, 4y	Hyperintensity of hippocampi and temporomesial regions (FLAIR); hyperintensity of BG (T2); hyperintensity of periventricular regions due to respiratory distress at birth (T2)	‐
Mackels et al^20^	M, 37y	Inferior vermian hypoplasia, corpus callosum dysgenesis (T1, FLAIR); hypointensity of GP and SN (T2*W‐GRE)	Calcifications of caudate nuclei, subtle calcifications of the GP
Jacobson et al^21^	F, 41y	Normal	‐
Dayan et al^22^	III‐7 (M, 46y)	Normal	No calcifications
	III‐10 (M, 47y)	Normal	Small calcifications of BG
Geoffre et al^7^	M, 49y	Hypointensity of GP, SN and dentate nuclei; mild frontal atrophy (FLAIR, SWI)	‐
	F, 52y	Normal	‐
Present case	M, 57y	Hypointensity of GP and SN (T2*); diffuse cortical atrophy (T1)	Cortical atrophy, no calcifications

Case reports were identified through a structured literature search of PubMed, Google Scholar, and Embase, retrieved through a literature search of major medical databases (PubMed, Scholar, Embase) covering all publications between 2010 up to 2025. Abbreviations: BG: basal ganglia; GP: globi pallidi; SN: substantia nigra; M: male; F: female; y: years; CT: Computed Tomography; MRI: Magnetic Resonance Imaging; FLAIR: Fluid‐Attenuated Inversion Recovery; GRE: Gradient Recalled Echo; SWI: Susceptibility‐Weighted Imaging. References are reported in Supplementary material.

Only two patients showed mild cortical atrophy, which was limited to the frontal region in one male patient,^7^ while it was diffuse with predominance in the left parietal lobe in our case. Follow‐up imaging after 5 years demonstrated that the asymmetric cortical atrophy remained stable, suggesting a developmental origin rather than a neurodegenerative process. This hypothesis is supported by the role of *RAB39B* in neuronal development, as the gene is highly expressed in the cortex, thalamus, basal ganglia and substantia nigra^2^, ^8^, ^9^ and its protein product is implicated in neurite and synapse formation.^4^, ^10^

While iron accumulation and cortical atrophy can occur in other neurodegenerative disorders—such as Neurodegeneration with Brain Iron Accumulation and tauopathies—the clinical presentation and progression in *RAB39B*‐related parkinsonism are clearly distinct, allowing for differential diagnosis.

A detailed assessment of brain imaging in a larger number of patients is needed to better define the spectrum and underlying pathology of brain defects in *RAB39B*‐associated parkinsonism.

## Author Roles

(1) Research project: A. Conception, B. Organization, C. Execution; (2) Statistical Analysis: A. Design, B. Execution, C. Review and Critique; (3) Manuscript: A. Writing of the first draft, B. Review and Critique.

L.G., E.B.: 1A, 1B, 1C, 3A.

S.N.: 1C, 3B.

A.P., S.G., S.B., S.C.: 1A, 3B.

M.A.: 1A, 1B, 1C, 3B.

E.M.V.: 1A, 1B, 3B.

## Disclosures

**Ethical Compliance Statement:** Patient informed consent was obtained for this work. The approval of an institutional review board was not required. We confirm that we have read the Journal's position on issues involved in ethical publication and affirm that this work is consistent with those guidelines.

**Funding Sources and Conflict of Interest:** The authors report no sources of funding or conflicts of interest with regards to the present topic.

**Financial Disclosures for the previous 12 months:** MA has received consulting fees or speaking honoraria from Bial, and research grants from Italian Ministry of Health. EMV has received research grants from the Italian Ministry of Health, Telethon Foundation and CARIPLO Foundation. The remaining authors declare that no sources of funding and no conflicts of interest.

## Supporting information


**Data S1.** Supporting Information.

## Data Availability

The data that support the findings of this study are available from the corresponding author upon reasonable request.
