# Supplementary material for: Asymmetric Parietal Cortical Atrophy in a Patient with RAB39B ‐Associated Parkinsonism
Source: Mov Disord Clin Pract. 2025 Aug 26;13(2):548–52. doi: 10.1002/mdc3.70310 (PMC13020572; doi:10.1002/mdc3.70310)
Supplement: Supplementary file 3 — Data S1. Supporting Information. [file MDC3-13-548-s001.docx]

# **Supplementary Material**

## **Supplementary Table 1. Clinical features of the patient at first neurological assessment and after 5 years.**

| **Clinical features** | **Baseline (52 years)** | **Follow-up (57 years)** |
| --- | --- | --- |
| MDS-UPDRS I | 0 | 21 |
| MDS-UPDRS II | 14 | 30 |
| MDS-UPDRS III | 18 (ON MED) | 53 (ON MED) |
| MDS-UPDRS IV | 0 | 6 |
| H&Y | 2 | 3 |
| LEDD (mg) | 300 | 757 |
| Q.I. | 53 | 53 |
| MMSE | 25 | 25 |
| hyposmia | not reported | yes (subjective) |
| pain | no | twinges, cramps, tingles |
| sleep disorders | no | insomnia, daytime sleepiness |
| mood disorders | no | depression, apathy, anxiety |
| autonomic symptoms | no | constipation, urge incontinence, hypotension/presyncope |
| impulsive compulsive disorders | no | no |
| drooling and dysphagia | no | yes |
| hallucinations / psychosis | no | no |

## **Supplementary references**

11. Vanmarsenille L, Giannandrea M, Fieremans N, et al. Increased dosage of RAB39B affects neuronal development and could explain the cognitive impairment in male patients with distal Xq28 copy number gains. Hum Mutat. 2014;35(3):377-383. doi:10.1002/humu.22497

12. Wilson GR, Sim JC, McLean C, et al. Mutations in RAB39B cause X-linked intellectual disability and early-onset Parkinson disease with α-synuclein pathology. Am J Hum Genet. 2014;95(6):729-735. doi:10.1016/j.ajhg.2014.10.015

13. El-Hattab AW, Schaaf CP, Fang P, et al. Clinical characterization of int22h1/int22h2-mediated Xq28 duplication/deletion: new cases and literature review. BMC Med Genet. 2015;16:12. doi:10.1186/s12881-015-0157-2

14. Lesage S, Bras J, Cormier-Dequaire F, et al. Loss-of-function mutations in RAB39B are associated with typical early-onset Parkinson disease. Neurol Genet. 2015;1(1):e9. doi:10.1212/NXG.0000000000000009

15. Güldner M, Schulte C, Hauser AK, Gasser T, Brockmann K. Broad clinical phenotype in Parkinsonism associated with a base pair deletion in RAB39B and additional POLG variant. Parkinsonism Relat Disord. 2016;31:148-150. doi:10.1016/j.parkreldis.2016.07.005

16. Shi CH, Zhang SY, Yang ZH, et al. A novel RAB39B gene mutation in X-linked juvenile parkinsonism with basal ganglia calcification. Mov Disord. 2016;31(12):1905-1909. doi:10.1002/mds.26828

17. Ciammola A, Carrera P, Di Fonzo A, et al. X-linked Parkinsonism with Intellectual Disability caused by novel mutations and somatic mosaicism in RAB39B gene. Parkinsonism Relat Disord. 2017;44:142-146. doi:10.1016/j.parkreldis.2017.08.021

18. Ballout RA, Dickerson C, Wick MJ, et al. Int22h1/Int22h2-mediated Xq28 duplication syndrome: de novo duplications, prenatal diagnosisdiagnoses, and additional phenotypic features. Hum Mutat. 2020;41(7):1238-1249. doi:10.1002/humu.24009

19. Santoro C, Giugliano T, Bernardo P, et al. A novel RAB39B mutation and concurrent de novo NF1 mutation in a boy with neurofibromatosis type 1, intellectual disability, and autism: a case report. BMC Neurol. 2020;20(1):327. doi:10.1186/s12883-020-01911-0

20. Mackels L, Moïse M, Depierreux F. Multimodal imaging of a patient with RAB39B mutation. Neuroradiology. 2022;64(3):621-625. doi:10.1007/s00234-021-02882-w

21. Jacobson JR, Piat C, Aksamit AJ, Patane' G, Ross OA, Savica R. Novel RAB39B loss-of-function mutation in patient with typical early-onset Parkinson's disease. Parkinsonism Relat Disord. 2024;123:106038. doi:10.1016/j.parkreldis.2024.106038

22. Dayan R, Shkedi Rafid S, Baker Erdman H, et al. Novel RAB39B Mutation Causes Parkinsonism in Males with Developmental Disorder. Mov Disord Clin Pract. 2024;11(3):306-308. doi:10.1002/mdc3.13953
